# Surgical outcome of Descemet’s stripping automated endothelial keratoplasty for bullous keratopathy secondary to argon laser iridotomy

**DOI:** 10.1007/s00417-012-1927-6

**Published:** 2012-01-28

**Authors:** Masatoshi Hirayama, Takefumi Yamaguchi, Yoshiyuki Satake, Jun Shimazaki

**Affiliations:** 1 Department of Ophthalmology, Ichikawa General Hospital, Tokyo Dental College, 5-11-13, Sugano, Ichikawa, Chiba 272-8513 Japan; 2 Department of Ophthalmology, Keio University School of Medicine, Tokyo, Japan

**Keywords:** Descemet’s stripping automated endothelial keratoplasty, Argon laser iridotomy, Fuchs’ dystrophy, Pseudophakic bullous keratopathy, Posterior lamellar keratoplasty

## Abstract

**Background:**

To report the 6-month clinical outcome of Descemet’s stripping automated endothelial keratoplasty (DSAEK) for bullous keratopathy (BK) secondary to argon laser iridotomy (ALI), and compare the results with those of DSAEK for pseudophakic bullous keratopathy (PBK) or Fuchs’ endothelial dystrophy (FED).

**Methods:**

A total of 103 patients (54 with ALI, 28 with PBK, 21 with FED) undergoing DSAEK were retrospectively analyzed. Simultaneous cataract surgery was performed in 37 patients with ALI and 13 with FED. Preoperative ocular conditions, best spectacle-corrected visual acuity (BSCVA), spherical equivalent refraction (SE), induced astigmatism, keratometric value, endothelial cell density (ECD), and complications were determined over 6 months postoperatively.

**Results:**

Mean axial length in the ALI group (21.8 ± 0.8 mm) was significantly shorter than that in the FED (*P* = 0.02) or PBK groups (*P* = 0.003). Severe corneal stromal edema (*n* = 6), advanced cataract (*n* = 10), posterior synechia (*n* = 3), poor mydriasis (*n* = 5), and Zinn zonule weakness (*n* = 1) were found only in the ALI group. A significant improvement was observed in postoperative BSCVA in all groups. No significant difference was observed in BSCVA, SE, induced astigmatism, keratometric value, ECD, or complications among the three groups.

**Conclusions:**

Descemet’s stripping automated endothelial keratoplasty for BK secondary to ALI showed rapid postoperative visual improvement, with similar efficacy and safety to that observed in DSAEK for PBK or FED.

## Introduction

**Fig. 1:**
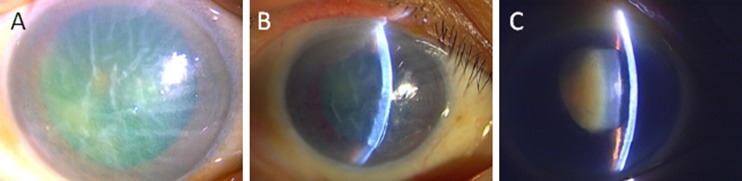
Slit-lamp photographs of three patients in ALI group taken preoperatively (a, b, c). Two cases (a, b) had severe corneal stromal edema with Descemet membrane fold with hard cataract. One case (c) had mild corneal stromal edema and rock-hard cataract with Zinn zonule weakness and poor mydriasis. DSAEK with cataract surgery had been performed safely on all patients

The cause of bullous keratopathy (BK), one of the main reasons for corneal transplantation worldwide, differs by region. For example, in addition to cataract surgery, Fuchs’ dystrophy is a major cause of BK in western countries [1]. In Japan, on the other hand, argon laser iridotomy (ALI) is the second most common cause for BK according to a recent national survey [2, 3]. Bullous keratopathy secondary to ALI (ALI-BK) can occur long after ALI, and both eyes with angle-closure glaucoma and those that have undergone prophylactic ALI may be affected. Descemet’s stripping automated endothelial keratoplasty (DSAEK), a lamellar corneal surgical procedure, allows selective replacement of the posterior layers of the cornea in the treatment of BK [4, 5]. The advantages of DSAEK over conventional penetrating keratoplasty include the need for only a small incision to be made, maintenance of structural integrity of the cornea, rapid visual recovery, and minimal induction of astigmatism [6–8]. However, when DSAEK is performed for ALI-BK, several challenges arise. These eyes characteristically have shallow anterior chambers which may render anterior chamber surgical maneuvers more difficult and risky. Therefore, DSAEK for ALI-BK is often technically challenging, even for well-experienced surgeons [9]. However, hitherto, reports on the surgical outcome of DSAEK for ALI-BK have only involved small patient samples [9, 10]. The incidence and management of the intra- and postoperative complications and visual outcomes of DSAEK for ALI-BK remain largely unknown. The aim of this study was to investigate the 6-month clinical outcome of DSAEK for ALI-BK, and to compare with those undergone DSAEK for other causes of BK (Fig. 1).

## Patients and methods

### Patients

The medical records of all consecutive patients undergoing DSAEK for BK resulting from ALI, Fuchs’ endothelial dystrophy (FED), or pseudophakic bullous keratopathy (PBK) between April 2007 and December 2010 at Tokyo Dental College Ichikawa General Hospital were retrospectively reviewed (Table 1). Patients were excluded from the analysis because of the following reasons; combined causes suspected such as ALI and FED (three cases), previous history of penetrating keratoplasty (eight cases), macular dysfunction due to previous retinal detachment (two cases) or Axenfeld-Rieger syndrome (one case), and end-stage glaucoma without central visual field (one case), or BK due to birth injury with corneal stromal opacity (two cases). The present study adhered to the tenets of the Declaration of Helsinki.

**Table 1:**
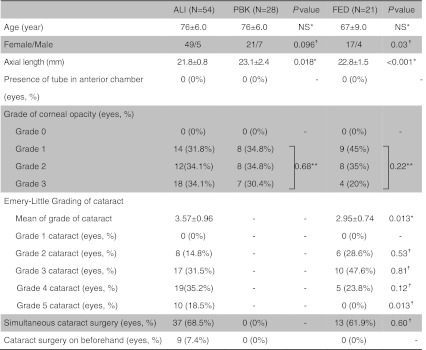
Patient demographics

^*^Mann–Whitney U test

**χ ^2^ test ^☨^ Fisher exact test

### Surgical procedures and postoperative treatment

After sub-Tenon or retrobulbar injection of 2% lidocaine, a 5.0-mm temporal or superior corneoscleral incision was made (details in Table 2). An anterior-chamber maintenance cannula was inserted for paracentesis. Descemet’s membrane stripping was performed with a diameter corresponding to the graft size, using a reverse-bent Sinsky hook (ASICO, Westmont, IL, USA) and an epithelial trephine marker. In most cases, the graft size was 8.0 mm, as shown in Table 2. Apart from seven eyes (13%) in the ALI group and four eyes (14%) in the PBK group in which nDSAEK were performed [10], the recipient’s endothelium and Descemet’s membrane were carefully removed by forceps. Pre-cut donor grafts were trephinated and the endothelial surface of the lenticle coated with a small amount of viscoelastic material (Viscoat®, Alcon, Fort Worth, TX, USA). Donor tissue was gently inserted into the anterior chamber using a Busin glide (ASICO, Westmont, IL, USA)/IOL glide and Shimazaki DSAEK forceps (Inami, Tokyo, Japan). Pull-through technique was used to insert the donor graft, except in six eyes (three eyes in the ALI group, two eyes in the PBK group and one eye in the FED group) in which the folding technique was used. Air was carefully injected into the anterior chamber to unfold the graft. Fluid from between the recipient’s stroma and the graft was drained via small incisions in the midperipheral recipient cornea. Ten minutes after air injection, most of the air was replaced with balanced salt solution (BSS plus®, Alcon, Fort Worth, TX, USA). At the end of the procedure, subconjunctival tobramycin 4 mg (Tobracin®, J-Dolph, Shiga, Japan) and betamethasone 0.4 mg (Rinderon®, Shionogi, Osaka, Japan) were administered. In patients with significant lens opacity, standard phacoemulsification and aspiration, and implantation of an intraocular lens were performed prior to DSAEK using the phaco-chop technique. Postoperative medication included 0.1% levofloxacin (Cravit®, Santen, Osaka, Japan) and 0.1% betamethasone sodium phosphate (Sanbetazon®, Santen, Osaka), starting at 5 times a day for 3 months and then tapering off thereafter.

**Table 2:** Details of surgical procedure of DSAEK in each groups

	ALI (*n* = 54)	PBK (*n* = 28)	FED (*n* = 21)
Descemet’s stripping endothelial keratoplasty (eyes, %)	47 (87%)	24 (86%)	21 (100%)
Non-Descemet’s stripping endothelial keratoplasty (eyes, %)	7 (13%)	4 (14%)	0 (0%)
Graft size (eyes)			
7.0 mm	1	0	0
7.5 mm	1	0	0
7.75 mm	1	1	1
8.0 mm	51	26	19
8.25 mm	0	0	1
8.5 mm	0	1	0
Location of corneoscleral incision (eyes, %)			
Temporal	51 (94%)	23 (82%)	20 (95%)
Superior	3 (6%)	5 (18%)	1 (5%)
Corneal epithelial removal (eyes, %)	13/54 (24%)	1/28 (3.6%)	4 (19%)
Trypan blue staining (eyes, %)	3/37 (8.1%)	0 (0%)	0 (0%)
Multiple sphincterotomy (eyes, %)	3/37 (8.1%)	0 (0%)	0 (0%)

### Examinations

Best spectacle-corrected visual acuity (BSCVA), spherical equivalent (SE), induced astigmatism [11], keratometric value (K value), and endothelial cell density (ECD) were measured pre- and postoperatively at 1, 3 and 6 months. Preoperative corneal opacity was semi-quantitatively graded using slit-lamp biomicroscopy as follows: grade 0: clear and normal; grade 1: slightly hazy, but iris cleft visible; grade 2: iris cleft difficult to identify; grade 3: iris cleft impossible to identify. Cataracts were graded according to the Emery–Little classification. Poor preoperative mydriasis was defined as a pupil diameter of less than 5 mm after instillation of tropicamide and phenylephrine 3 times per 10 minutes. Decimal values of BCSVA were converted to logarithm for statistical analysis. Endothelial cell density was measured using a specular microscope (SP-3000P®, Tomey, Nagoya, Japan). Intra- and postoperative complications were also recorded.

### Statistical analysis

The Mann–Whitney U, Wilcoxon, Kruskal–Wallis, χ^2^ tests and Fisher’s exact test were used for the statistical analysis. A *P* value of less than 0.05 was considered to indicate statistical significance. All statistical analyses were performed with the SSRI software (SSRI Co. Ltd., Tokyo, Japan).

## Results

### Preoperative ocular conditions

Table 1 summarizes the demographic data on the 103 eyes with BK analyzed in this study, which included 54 with ALI-BK (ALI group), 28 with PBK (PBK group), and 21 with FED (FED group). The proportion of eyes that had simultaneous cataract surgery is shown in Table 1. Mean axial length was significantly smaller in ALI group than in FED (*P* = 0.02) or PBK groups (*P* = 0.0003, Mann–Whitney U test). As shown in Table 1, the grade of corneal opacity was similar in each group. However, severe corneal stromal edema with descemet’s membrane fold occurred in six eyes (11%) in ALI group. Moreover, posterior synechia (*n* = 3) and poor mydriasis (*n* = 5) occurred more frequently in ALI group. Mean grade of cataract was significantly higher in ALI group than in FED group, with grade 5 cataract found only in ALI group (Table 1). Higher than grade 4 cataract occurred significantly more often in the ALI group than in the FED group (*P* = 0.023; Fisher exact test). As shown in Table 2, various types of intraoperative manipulation, including corneal epithelium removal, trypan blue-assisted phacoemulsification and multiple sphincterotomy, were required only in ALI group.

### BSCVA, induced astigmatism, SE and K value

All eyes were followed up for a minimum of 6 months postoperatively. Clarity was maintained in the donor graft at postoperative month 6 in 50 eyes (92.6%) in ALI group, 26 eyes (92.9%) in PBK group, and 20 eyes (95.2%) in FED group. Table 3 summarizes BSCVA at preoperative and postoperative months 1, 3 and 6. With regard to postoperative BSCVA, 53 eyes (98.1%) in ALI group, 26 eyes (92.9%) in PBK group and 19 eyes (90.4%) in FED group showed improved BSCVA. Forty-four eyes (81.5%) in ALI group, 19 eyes (67.9%) in PBK group and 14 eyes (66.7%) in FED group achieved a BSCVA of 20/40 or better at 6 months. A significant improvement was observed in BSCVA from 1 month after DSAEK in each group (*P* < 0.0001 in ALI group, *P* = 0.0006 in PBK group, and *P* = 0.0004 in FED group; Wilcoxon test). In ALI and PBK groups, BSCVA improved significantly from 1 month to 3 months postoperatively (*P* < 0.0001 in ALI group and *P* = 0.0065 in PBK group; Wilcoxon test). Table 4 shows the results of refractive data. No significant difference was observed in induced astigmatism throughout the postoperative observation period in each group. No significant difference was observed in SE between the preoperative and postoperative 1-month values in any group. A significant difference was observed in SE between postoperative month 1 and 6 in FED group (*P* = 0.046, Wilcoxon test). No significant differences were observed in preoperative and 1-, 3-, and 6-month postoperative K values in any group.

**Table 3:** The average of best spectacle-corrected visual acuity (BSCVA)

BSCVA (LogMAR ± SD)	ALI (range)	PBK (range)	FED (range)	*P* value
Preoperative	1.27 ± 0.64 (0.16 to 3)	1.41 ± 0.59 (0.52 to 2.7)	0.88 ± 0.49 (0.3 to 2)	
1 m	0.60 ± 0.63 (0.16 to 3)	0.78 ± 0.64 (0.3 to 2.7)	0.44 ± 0.46 (0.16 to 2)	NS*
3 m	0.43 ± 0.57 (0 to 2.7)	0.65 ± 0.67 (0.1 to 2.7)	0.39 ± 0.44 (0.05 to 2)	NS*
6 m	0.34 ± 0.60 (−0.08 to 2.7)	0.50 ± 0.65 (0 to 2.7)	0.21 ± 0.30 (0 to 1)	NS*
Eyes showing improved BSCVA (eyes)	53 (98.1%)	26 (92.9%) P = 0.27^☨^	19 (90.4%) P = 0.19^☨^	
Postoperative BSCVA >20/40 (eyes)	44 (81.5%)	14 (66.7%) P = 0.18^☨☨^	19 (67.9 %) P = 0.22^☨☨^	

*Kruskal–Wallis test comparing the difference among three groups

☨ Fisher’s test comparing the number of eyes showing improved BSCVA between in ALI group and PBK group or FED group

☨☨ Fisher’s test comparing the number of eyes showing postoperative BSCVA >20/40 between in ALI group and PBK group or FED group

**Table 4:** Graft survival rate and refractive data

	ALI	PBK	FED	*P* value
Graft survival rate at postoperative 6 m (eyes, %)	50 (92.6%)	26 (92.9%)	20 (95.2%)	
Induced astigmatism (D)				
1 m	1.4 ± 1.2	2.5 ± 2.1	2.3 ± 1.4	0.007*
3 m	1.5 ± 1.1	2.3 ± 1.1	2.0 ± 1.6	0.036*
6 m	1.5 ± 1.0	1.8 ± 0.7	1.7 ± 1.3	NS*
Spherical equivalence (D)				
Preoperative	0.69 ± 2.6	−0.74 ± 1.86	−0.25 ± 1.5	NS*
1 m	0.31 ± 1.6	−0.48 ± 2.2	−0.39 ± 2.2	NS*
3 m	0.17 ± 1.2	−0.94 ± 2.0	1.6 ± 5.26	NS*
6 m	−0.15 ± 0.78	−0.94 ± 2.0	−2.7 ± 4.3	NS*
Keratometric value				
Preoperative	44.5 ± 2.4	43.8 ± 2.0	44.5 ± 2.3	NS*
1 m	43.3 ± 2.4	43.3 ± 2.2	44.0 ± 2.2	NS*
3 m	44.0 ± 2.2	43.6 ± 1.9	43.5 ± 2.6	NS*
6 m	44.0 ± 2.1	43.7 ± 2.1	43.8 ± 1.4	NS*

Data were presented as mean ± SD. *Kruskal–Wallis test compared the difference among three groups

### Endothelial cell loss

Table 5 summarizes ECD of donor preoperatively and ECD at postoperative month 1, 3 and 6. Preoperative ECD significantly decreased 1 month after DSAEK (*P* < 0.0001 in ALI group, *P* = 0.043 in PBK group, and *P* = 0.012 in FED group; Wilcoxon test). In addition, postoperative ECD significantly decreased from month 3 (1174 ± 361 cells/mm^2^) to month 6 (944 ± 386 cells/mm^2^) in PBK group (*P* = 0.017; Wilcoxon test), although no rejection episode occurred between 3 and 6 months in this group.

**Table 5:** Endothelial cell density (ECD)

ECD(/mm^2^ ± SD)	ALI	PBK	FED	*P* value
Donor preoperatively	2339 ± 284	2600 ± 341	2701 ± 332	NS*
1 m	1360 ± 467	1023 ± 260	1158 ± 487	NS*
3 m	1217 ± 485	1174 ± 361	1388 ± 612	NS*
6 m	1124 ± 427	944 ± 386	1230 ± 560	NS*

*Kruskal–Wallis test comparing endothelial cell density among three groups

### Complications

Complications are summarized in Table 6. No significant differences were observed in incidence of intra- or postoperative complications among the three groups. Three eyes out of 37 eyes (8.1%) in ALI group showed posterior capsule rupture intraoperatively. In two of these three eyes, an intraocular lens was inserted into the capsular bag, while in the remaining eye the lens was fixed to the sulcus. Twelve out of 103 eyes (11.5%) (six eyes in ALI group, three eyes in PBK group and three eyes in FED group) showed dislocation of the donor corneal lenticle at day 1 postoperatively, with each undergoing successful reattachment with one or a pair of air bubble tamponades. Pupillary block glaucoma secondary to anterior chamber air bubble occurred in three out of 103 eyes (2.9%) (one eye in ALI group and two eyes in PBK group) on the day of surgery, and was successfully treated by air removal. One out of 103 eyes (0.97%) (PBK group) developed acute graft rejection characterized by mild inflammation in the anterior chamber and keratic precipitates on the donor endothelium. In this case, graft rejection was treated with intensive topical and intravenous corticosteroid therapy. No case underwent re-keratoplasty within 6 months. Postoperative ocular hypertension developed in six eyes (three eyes in ALI group, one eye in PBK group and two eyes in FED group) and was treated with anti-glaucoma eye drops. Cystoid macular edema was found in one eye in ALI group, and was treated with intensive instillation of 0.1% diclofenac sodium (Diclod®, Wakamoto Pharmaceuticals, Tokyo, Japan).

**Table 6:** Complications

		ALI	PBK	*P* value*	FED	*P* value*	Total
Intraoperative	Posterior capsule rupture (eyes, %)	3 (8.1%)	0 (0%)	0.54	0 (0%)	0.56	3 (2.9%)
Postoperative; early	Dislocation (eyes, %)	6 (11%)	3 (11%)	1	3 (14%)	0.70	12 (12%)
	Pupillary block (eyes, %)	1 (1.9%)	2 (7.1%)	0.27	0 (0%)	1	3 (2.9%)
Postoperative; chronic	Rejection (eyes, %)	0 (0%)	1 (3.6%)	1	0 (0%)	1	1 (0.97%)
	Ocular hypertension (eyes, %)	3 (5.5%)	1 (3.6%)	1	2 (9.5%)	0.61	6 (5.8%)
	Cystoid macular edema (eyes, %)	1 (1.9%)	0 (0%)	1	0 (0%)	1	1 (0.97%)

*Fisher exact test compared with ALI group

## Discussion

We evaluated the 6-month clinical outcome of Descemet’s stripping automated endothelial keratoplasty (DSAEK) for BK secondary to ALI and compare the results with those of DSAEK for pseudophakic bullous keratopathy (PBK) or Fuchs’ endothelial dystrophy (FED). We demonstrated that DSAEK for BK secondary to ALI showed rapid postoperative visual improvement, with similar efficacy and safety to that observed in DSAEK for PBK or FED.

Bullous keratopathy secondary to ALI is becoming increasingly common in Asian countries, especially in Japan [2, 3, 12–15], where a national survey revealed that ALI-BK accounted for approximately one-fourth of BK cases undergoing keratoplasty. Most cases of ALI-BK develop long after laser iridotomy, in which an argon laser is used [2]. However, the underlying mechanism of ALI-BK remains unclear. Several hypotheses have been postulated, including increased temperature in the local aqueous humor [16], high energy delivered during ALI breakdown of the blood–aqueous barrier, and change in aqueous humor fluid dynamics [17, 18].

This study demonstrated that surgery for ALI-BK is technically challenging, mainly due to the small size of the eyeball shallow anterior chamber and challenging simultaneous cataract surgery in ALI group. In the present study, the mean axial length of eyes receiving DSAEK for ALI-BK was less than 22 mm. In addition, advanced cataract is often associated with this disorder, and physicians are reluctant to perform surgery due to decreased endothelial density. Moreover, a shortage of donor grafts in Japan often gives rise to delayed keratoplasty, which makes the procedure even more challenging. There were also associated problems such as poor mydriasis and weak Zinn zonule in some cases. To overcome difficulties in surgery, various supporting surgical procedures are used, and simultaneous cataract surgery. Epithelial removal technique was performed in case of invisible anterior chamber by strong corneal epithelial edema. In addition, trypan blue staining was performed to stain anterior capsule for continuous curvilinear capsulorhexis (CCC) in the case of severe cataract. In this study, all trypan blue staining was combined with epithelial removal procedure. The postoperative outcome in eyes with ALI-BK was comparable to other groups, suggesting that combined DSAEK and cataract surgery can be performed without severe complications with the above-mentioned surgical technique in most cases.

In this study, the average BSCVA 6 month after DSAEK for ALI-BK was 20/29 with low induced astigmatism. Seven eyes (12.9%) achieved postoperative BSCVA of 20/20 or better. Clarity was maintained in the donor graft at postoperative month 6 in 50 eyes (92.6%). No case underwent re-keratoplasty within 6 months. These results are comparable with those of Gorovoy [5], Koenig [6], and Kobayashi [9] (Table 7). Endothelial cell loss was 52% at postoperative 6 months, similar to 6-month data reported previously [6]. Postoperative endothelial loss after DSAEK for ALI-BK was not significantly different from that in the other two groups. We were concerned about the possibility that the ALI group may experience a faster decrease in ECD than the other groups after DSAEK, because of the pathogenesis mechanism of ALI. However, the results of this study showed that the 6-month clinical outcomes of ECD in DSAEK for ALI-BK were comparable with those of DSAEK for BK caused by FED or PBK [20, 21]. One of the explanations may be that simultaneous cataract surgery eliminates the cause of endothelial cell damage by deepening the anterior chamber.

**Table 7:** Comparison of clinical outcome with previous reports

Author Journal	Gorovoy et al. Cornea, 2006 [5]	Koenig et al. Cornea, 2007 [6]	Koenig et al. Ophthalmology, 2007 [7]	Bahar et al. Ophthalmology, 2008 [8]	Kobayashi et al. Cornea, 2008 [9]	Kobayashi et al. Am J O, 2008 [10]	Price et al. Ophthalmology, 2010 [19]	Hirayama et al.
Operation	DSAEK	DSAEK	DSAEK	DSAEK	DSAEK	nDSAEK	DSAEK	DSAEK/ nDSAEK
Study design	Retrospective	Prospective	Prospective	Prospective	Prospective	Prospective	Prospective	Retrospective
Number of eyes (eyes)	16	34	26	45	14	6	173	54
Disease (eyes)	FED 9	FED 11	FED 12	FED 28	ALI-BK 14	ALI-BK 6	FED 147	ALI-BK
	PBK 7	P/ABK 23	P/ABK 14	PBK 12			P/ABK 23	
				ICE syndrome 2			Other endothelial failure 3	
				Failed graft 3				
M:F	7:9	6:28	6:20	20:25	1:13	2:4	69:104	5:49
Mean age (years)	66	73.8	75.9	70.2	74.2	74.5	72	76
Follow-up time	1 year	6 months	3 months	9.8 months	228 ±132 days	6 months	1 year	6 month
Postoperative BSCVA	20/40 or better (except three eyes with macular scar, optic dystrophy, primary graft failure)	Mean 20/42 (62% achieved 20/40 or better)	Mean 20/45	Mean 20/44	20/40 or better (23% achieved 20/20)	More than 20/32 (33% achieved 20/20)	–	Mean 20/29 (13% achieved 20/20)
Postoperative SE (D)	–	0.97	0.82	0.96	–	–	–	−0.15
Postoperative astigmatism (D)	–	1.80	2.12	1.36	0.53	0.85	–	1.5
Postoperative ECD (/mm^2^)	1,714 (except one eye of primary graft failure)	1,396	–	1,735	1,654	2,391	1,743	1,124
Cell loss	41%	50%	–	36%	45%	26%	38%	52%
Complications during operation (eyes)	–	–	–	–	Vitreous prolapse 2 (14%)	None	–	Posterior capsule rupture 3 (5.5%)
Postoperative complications (eyes)			–					
Graft failure	1 (6%)	3 (9%)	–	1 (2%)	–	0 (0%)	–	–
Dislocaton	4 (25%)	9 (27%)	9 (35%)	7 (16%)	2 (14%)	1 (17%)	10 (6%)	6 (11%)
Acute rejection	–	6 (18%)	3 (12%)	1 (2%)	–	–	9 (5%)	0 (0%)
Elevated IOP	–	–	–	3 (7%)	–	–	27 (16%)	3 (6%)
Papillary block	–	1 (3%)	1 (4%)	–	–	0 (0%)	0 (0%)	1 (2%)
Others				Interface opacity 2 (4%)		Subclinical endothelial rejection 1 (17%)	Retinal detachment 1(<1%)	CME 1 (2%)
				CME 1 (2%)			Anterior synechiae 2 (1%)	

DSAEK; Descemet’s stripping automated enthothelial keratoplasty, nDSAEK; non-Descemet’s stripping automated enthothelial keratoplasty, FED; Fuchs’ endothelial dystrophy, PBK; pseudophakic bullous keratopathy, ABK; aphakic bullous keratopathy, ALI-BK; bullous keratopathy secondary to argon laser iridotomy, ICE syndrome; iridocorneal endothelial syndrome, SE; spherical equivalent, ECD; endothelial cell density, IOP; intraocular pressure, CME; cystoid macular edema

Our previous report on PK for ALI-BK showed a similar result in BSCVA to that of the present study (78.6% of patients achieved 20/40 or better); however, incidence of rejection (8.2%) and postoperative glaucoma (18.4%) was relatively high [22]. The refractive outcome at postoperative 6 months of our previous study indicated that PK for ALI-BK resulted in similar SE (the average was 0.19 ± 4.6 D) to the present study, and higher induced astigmatism (the average; 3.3 ± 2.4 D) (unpublished data). Although there was no report that showed postoperative refraction data of PK for ALI-BK in detail, PK usually results in unstable refraction as long as sutures are present, and even after their removal [23–26]. Bahar et al. reported the comparison of 12-month surgical outcomes of DSAEK and PK [8]. Stability of the refraction is a major advantage of all endothelial keratoplasty techniques as compared with PK. In current study, the postoperative refraction after DSAEK for ALI-BK was stable during follow-up terms.

In summary, this study of 6-month outcomes of DSAEK for BK secondary to ALI showed rapid postoperative visual improvement, with similar efficacy and safety to that observed in DSAEK for PBK or FED. Although many of the eyes in ALI group presented technical challenges during surgery, those challenges could be successfully managed by modification of the procedures and implements used.
